# Mechanism of motoneuronal and pyramidal cell death in amyotrophic lateral sclerosis and its potential therapeutic modulation

**DOI:** 10.1038/s41420-024-02055-7

**Published:** 2024-06-19

**Authors:** Bernát Nógrádi, Dóra Nógrádi-Halmi, Barbara Erdélyi-Furka, Zalán Kádár, Tamás Csont, Renáta Gáspár

**Affiliations:** 1 Department of Neurology, Albert Szent-Györgyi Health Centre, University of Szeged, Szeged, Hungary; 2 Euan MacDonald Centre for Motor Neuron Disease Research, University of Edinburgh, Edinburgh, UK; 3 Department of Biochemistry, Albert Szent-Györgyi Medical School, University of Szeged, Szeged, Hungary; 4 Interdisciplinary Centre of Excellence, University of Szeged, Szeged, Hungary; 5 Department of Anatomy, Histology and Embryology, Albert Szent-Györgyi Medical School, University of Szeged, Szeged, Hungary

**Keywords:** Necroptosis, Amyotrophic lateral sclerosis, Molecular neuroscience, Apoptosis, Autophagy

## Abstract

Amyotrophic lateral sclerosis (ALS) is a fatal neurodegenerative disorder clinically characterized by muscle atrophy and progressive paralysis. Loss of motoneurons and pyramidal cells is thought to be the center piece of the complex and multifaceted ALS pathology, however, the exact mechanisms laying behind motoneuronal cell death in the spinal cord and motor cortex are still unknown. It was originally proposed that apoptosis plays a fundamental role in motoneuronal demise, nonetheless, later it became clear that other forms of regulated cell death, including necroptosis, pyroptosis, ferroptosis, and autophagy-dependent cell death, may also contribute to motoneuron loss. Over the past years, multiple studies aimed to improve our understanding of the contributory role of these mechanisms as well as to offer novel targets for potential therapeutic interventions. The pharmacological inhibition of the ferroptotic pathway and the modulation of the autophagic machinery seem to have particularly promising effects, reducing motoneuron loss and slowing disease progression in transgenic models of ALS. Nevertheless, the potential beneficial effects of necroptosis-targeting interventions were mostly disproven in the latest studies. In this review we aim to summarize the current view on regulated cell death mechanisms that lead to motoneuronal and pyramidal cell degeneration in ALS and showcase their applicability as future drug targets.

## Facts

1. Motoneuronal death is a hallmark of amyotrophic lateral sclerosis.
2. Besides apoptosis, additional regulated cell death pathways were also implicated in ALS.
3. Targeting cell death pathways was shown to be beneficial in various ALS models.

## Open questions

1. Which cell death pathways contribute to motoneuronal and cortical pyramidal cell degeneration in ALS?
2. How concise is the activation of cell death pathways across different studies and various models of ALS?
3. Which pharmacological and genetic interventions brought tangible improvements in preclinical settings?

## Introduction

Amyotrophic lateral sclerosis (ALS) is a fatal neurodegenerative disorder, characterized by the selective loss of bulbar and spinal cord motoneurons and pyramidal Betz cells in the motor cortex. The resulting progressive muscle weakness, atrophy and paralysis leads to respiratory failure and death 2–5 years from the onset of the symptoms [1]. ALS is considered to be a rare disease, with a lifetime risk of 1:400 [2], nevertheless the number of ALS cases is projected to increase by 70% by 2040 [3].

Causative genetic mutations are detectable in 10–15% of ALS patients, including mutations on the superoxide dismutase 1 (*SOD1*), TAR DNA‐binding protein (*TARDBP*), and chromosome 9 open reading frame 72 (*C9orf72*) genes [4]. However, in the majority of cases the cause of the disease remains unclear. This is partially due to the complex and multifaceted pathophysiology of ALS, as protein aggregation [5], excitotoxicity [6], mitochondrial dysfunction [7], autophagy dysregulation [8] and neuroinflammation [9] are all key features of the unfolding motoneuronal degeneration. These destructive mechanisms eventually culminate in the loss of the alpha motoneurons and pyramidal Betz cells, which are the hallmarks of ALS pathology. It was originally proposed that motoneuronal death happens via apoptosis [10], however, later it was suggested that additional, yet unidentified cell death mechanisms might be present [11]. Our understanding of cell death mechanisms drastically expanded in the past years [12], as reflected by the novel nomenclature recommendation of cell death [13]. Emerging evidence suggests that besides apoptosis; necroptosis, pyroptosis, ferroptosis and autophagy-dependent cell death might all contribute to motoneuron and pyramidal cell loss. In this review, we summarize the supporting data for each of these cell death mechanisms and the current view on their therapeutic potential.

## Methodology

We conducted our search in the PubMed data base, up until 15 May 2024. Our search included the keywords “amyotrophic lateral sclerosis”, “ALS” or “motoneuron disease” and “apoptosis”, “caspase”, “necroptosis”, “RIPK”, “pyroptosis”, “inflammasome”, “NLRP3”, “ferroptosis”, “GPX4”, “autophagy”, or “autophagy-related cell death”, “LC3”. This search was supplemented with keywords “epigenetics”, “miRNA”, “therapy”, “modulation” and “treatment”.

## Apoptosis

**Fig. 1:**
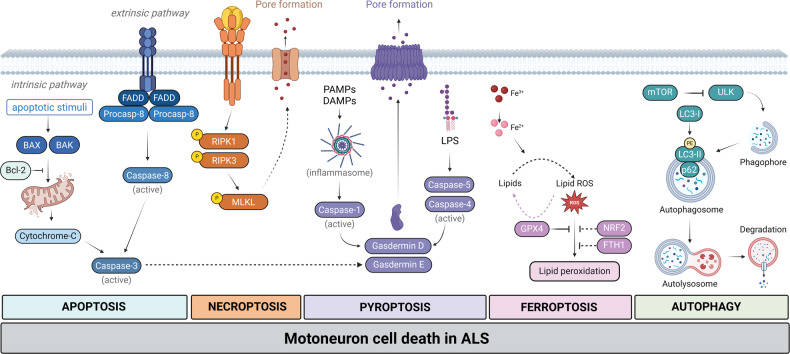
Overview of cell death pathways contributing to motoneuronal loss in ALS. BAX: Bcl-2 associated X; BAK: Bcl-2 homologous antagonist/killer; Bcl-2: B-cell lymphoma 2; FADD: FAS-Associated via Death Domain; RIPK: receptor-interacting protein kinase; MLKL: mixed lineage kinase domain like pseudokinase; PAMP: Pathogen-associated molecular patterns; DAMP: Danger-associated molecular patterns; LPS: lipopolysaccharide; ROS: reactive oxygen species; GPX4: glutathione peroxidase 4; NRF2: NF-E2–related factor 2 ; FTH1: ferritin heavy chain 1; mTOR: mammalian target of rapamycin; ULK: Unc-51-like kinase; LC3: light chain 3. The figure was created with BioRender.com.

Apoptosis, or programmed cell death (PCD) is described by characteristic morphological changes and energy-dependent biochemical mechanisms [14]. While PCD is an essential component of physiological cellular homeostasis, abnormal activation of apoptosis plays a crucial role in the pathomechanism of several neurodegenerative disorders [15]. The two major pathways involved in PCD are the intrinsic and extrinsic pathways (Fig. 1). The intrinsic pathway is a non-receptor mediated cell death mechanism, triggered by a wide range of signals (e.g., hypoxia, reactive oxygen species), resulting in cytochrome-C release from the mitochondria into the cytosol and consequent activation of the caspase cascade [16]. The extrinsic pathway relies on the activation of death receptors (e.g., FAS receptor) [17], also converging on the caspase cascade [18]. Both pathways induce characteristic morphological changes of the cell structure, such as cell shrinkage, chromatin condensation, micronuclei formation and cell fragmentation [19, 20].

In the early stages of rodent and human ALS research, apoptosis was considered to be a primary feature of motoneuronal degeneration. In 1999, the post-mortem analysis of the spinal cord of ALS patients revealed that the number of B-cell lymphoma 2 (Bcl-2) associated X (Bax) positive motoneurons was higher, indicative of apoptotic mechanisms [21]. This finding was supported by Martin, who reported an apoptotic-like morphology of spinal cord motoneurons in human post-mortem samples. The increased expression of proapoptotic Bax, Bcl-2 homologous antagonist/killer (Bak) and activation of effector caspase-3 was also detected in the spinal cord of ALS patients, while the antiapoptotic Bcl-2 was decreased, supporting the activation of the apoptotic pathway [10].

Multiple studies analyzed the expression of microRNAs (miRNA) regulating the expression of a wide range of apoptosis-related proteins, including, but not limited to elements of the caspase cascade, Bcl-2 family members and p53. These investigations revealed increased miRNA-155, miRNA-29a, miRNA-181a and decreased miRNA-22 levels in the spinal cord of SOD1 transgenic mice, resulting in an overall shift towards proapoptotic signaling [22–24]. However, as these miRNAs act on multiple targets, and may exert dual (i.e., positive, or negative) regulatory effects on a single pathway, further clarification is needed on their exact role in neuronal cell death. The altered epigenetic regulation of apoptotic signaling was further corroborated by post-mortem analysis of human spinal cord samples, which revealed aberrant DNA methylation driving neuronal apoptosis [25].

Regarding the downstream executive mediators, Li et al. reported caspase-1 and caspase-3 activation in the spinal cord of symptomatic SOD1^G93A^ mice (i.e., the most commonly used transgenic model of ALS), however, these changes were not observed in the presymptomatic stage. In the same study, a broad caspase inhibitor, zVAD-fmk, tendentiously prolonged the survival and significantly improved motor strength and coordination [26].

The activation of pro-apoptotic mechanisms has also been implicated in the motor cortex by various reports. The level of cleaved caspase-3 was increased pre-symptomatically in corticospinal pyramidal cells in SOD1^G93A^ mice [27], which was further corroborated by the accumulation of cleaved caspase-3 in pyramidal cells in the motor cortex of ALS patients [28]. Additionally, increased expression of pro-apoptotic regulator proteins Bak and Bax was also reported in the post-mortem motor cortex samples of ALS patients [10].

The involvement of the extrinsic apoptotic pathway in ALS pathology was indicated in an in vitro study, where primary motoneurons, isolated from various SOD1-mutant mice, were more susceptible to Fas-triggered cell death [29]. Somewhat controversially, glycogen synthase kinase-3β (GSK-3β) inhibitor treatment of SOD1^G93A^ mice seemed to induce a slight elevation in the activity of the extrinsic apoptotic pathway, while improving the motor activity and delaying the onset of symptoms. Nevertheless, the GSK-3β inhibitor failed to exert its beneficial effect in the symptomatic stage [30]. Various studies showed that minocycline can prevent the overactivation of apoptosis and could be beneficial in neurodegenerative diseases [31]. Zhu et al. reported that minocycline delayed the disease onset and prolonged the survival of SOD1^G93A^ mice, potentially via the direct inhibition of cytochrome-C release [32]. The beneficial effects of minocycline on disease progression were also corroborated in the SOD1^G37R^ transgenic mouse model of ALS [33]. Antagomir-mediated knockdown of miRNA-29a in the spinal cord and brain has been reported to delay disease onset and prolong the survival of SOD1^G93A^ mice [24]. Moreover, treatment with the oligonucleotide-based anti-miRNA-155 inhibitor has been shown to prolong the survival of SOD1^G93A^ mice [23].

**Fig. 2:**
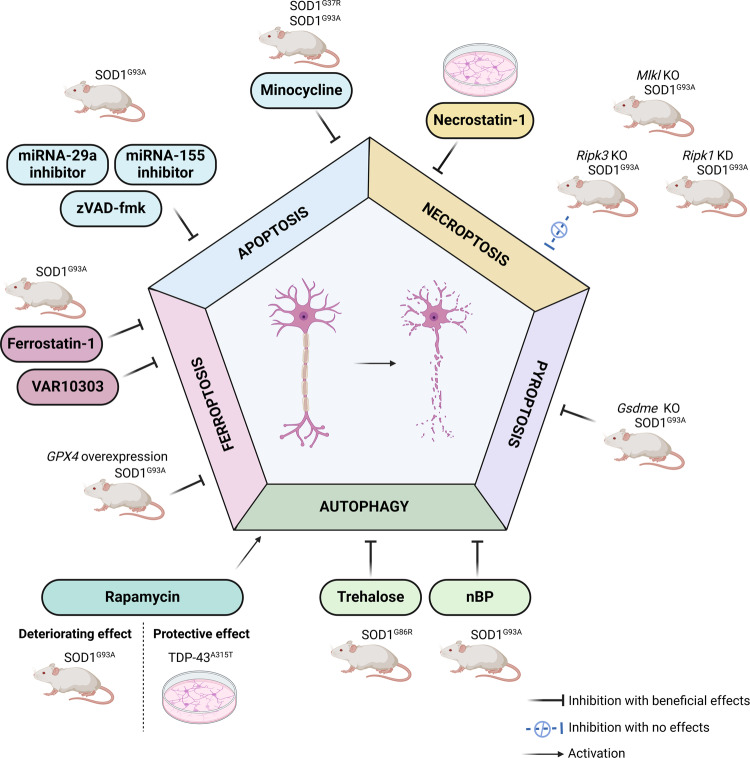
The effect of modulation of cell death pathway in different models of ALS. *GPX4*: glutathione peroxidase 4; *Ripk*: receptor-interacting protein kinase; *Mlkl*: mixed lineage kinase domain like pseudokinase; *Gsdme*: gasdermin E; nBP: n-butylidenephthalide; KO: knock-out; KD: knock-down. The figure was created with BioRender.com.

**Fig. 3:**
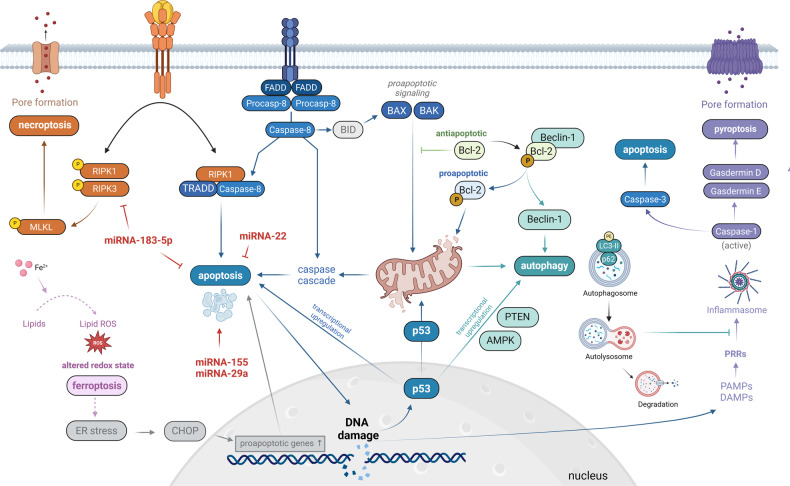
Interplay among cell death pathways contributing to neuronal degeneration in ALS. AMPK: adenosine monophosphate-activated protein kinase; Bax: Bcl-2 associated X; Bak: Bcl-2 homologous antagonist/killer; Bcl-2: B-cell lymphoma 2; Bid: BH3-interacting domain death agonist; CHOP: C/EBP homologous protein; DAMP: Damage-associated molecular patterns; ER: endoplasmic reticulum; FADD: Fas-associated death domain; GPX4: glutathione peroxidase 4; RIPK-1/-3: receptor-interacting protein kinase-1/-3; Mlkl: mixed lineage kinase domain like pseudokinase; PAMP: Pathogen-associated molecular pattern; PRR: Pattern recognition receptors; PTEN: Phosphatase and tensin homolog; ROS: reactive oxygen species; TRADD: TNFR1-associated death domain protein. The figure was created with BioRender.com.

In spite of these findings, multiple studies raised doubts about the involvement of apoptosis in motoneuronal death in ALS, partially due to uncertainty of interpreting PCD on the basis of morphological alterations (Table 1) [11, 34]. Regardless of this controversy, it is clear that the modulation of the apoptotic pathway via different mechanisms could only slightly slow the loss of spinal cord motoneurons (Fig. 2). Moreover, due to the robust increase in the understanding of various novel cell death mechanisms over the past decade, research focus has somewhat shifted from apoptosis to alternative cell death mechanisms in ALS, and to the potential interactions among these pathways (Fig. 3). Thus, in the following sections we discuss the involvement of necroptosis, pyroptosis, ferroptosis and autophagy in motoneuronal and pyramidal cell death.

**Table 1:** Reports indicating the mechanism of motoneuronal cell death in ALS.

	Disease model/sample type	Main findings (cell-/tissue-specific)	Ref.
Apoptosis	ALS patient samples	Bax, Bax/Bcl-2 ratio and DNA fragmentation ↑ (motoneurons)	[21]
	sporadic and familial ALS patient samples	increased Bax, Bak, reduced Bcl-2, elevated casp-3 activation, apoptotic morphology (motoneurons)	[10]
	SOD1^G93A^ mice	casp-1 and 3 activities ↑ (motoneurons)	[26]
	in vitro primary motoneuron culture isolated from SOD1^G93A, G85R, G37R^ mice	activation of Fas-mediated pathway ↑ (in vitro motoneuron culture)	[29]
	sporadic and familial ALS patient samples	Bax, Bak ↑, casp-3 ↑ (motor cortex)	[10]
	ALS patient samples	ssDNA and cleaved casp-3 ↑ (motor cortex)	[28]
	SOD1^G93A^ mice	cleaved casp-3 ↑ (motor cortex)	[27]
Necroptosis	SOD1^G93A^ mice	*Ripk1*, *Ripk3*, *Mlkl* mRNA ↑, RIPK1 protein ↑ (total spinal cord)	[39]
	OPTN KO mice	RIPK1, RIPK3, MLKL ↑ (total spinal cord)	[40]
	SOD1^G93A^ mice	pRIPK1 ↑ (total spinal cord)	[41]
	SOD1^G93A^ mice	RIPK1 ↑ (total spinal cord)	[42]
	ALS patient samples	no difference in RIPK1 (motor cortex)	[39]
	SOD1^G93A^ mice	no difference in RIPK1 (motor cortex)	[41]
	SOD1^G93A^ mice	no difference in RIPK1 (frontal cortex)	[42]
	ALS patient samples	no pRIPK1, pRIPK3, pMLKL detected (spinal cord and motor cortex)	[44]
	ALS patient samples	pMLKL ↑ (hippocampus)	[44]
Pyroptosis	SOD1^G93A^ mice spinal cord	NLRP3, casp-1, GSDMD ↑ (motoneurons)	[48]
	sporadic and C9orf72 ALS patient samples	casp-1, GSDMD ↑ (total spinal cord)	[52]
	SOD1^G93A^ mice spinal cord	GSDME ↑ (total spinal cord)	[53]
	sporadic and C9orf72 ALS patient samples	cleaved GSDMD, NLRP3 ↑ (motor cortex)	[52]
	mouse cortical neurons transfected with TDP-43	GSDME ↑ (in vitro TDP-43 model)	[53]
Ferroptosis	SOD1^G93A^ mice spinal cord	GSH, GPX4 ↓ (total spinal cord)	[57]
	SOD1^G93A^ mice spinal cord	morphological signs of ferroptosis (motoneurons)	[57]
	SOD1^G93A^, TDP-43^Q331K^ and C9orf72-500 mice	GPX4 levels ↓ (total spinal cord)	[58]
	SOD1^G93A^ mice	FTH1 and NRF2 levels ↓ (total spinal cord)	[58]
	sporadic and familial ALS patient samples	GPX4 levels ↓ (total spinal cord)	[58]
	sporadic ALS patient samples	iron accumulation (motoneurons)	[59]
	SOD1^G93A^ mice	GSH, GPX4 ↓ and morphological signs of ferroptosis (motor cortex)	[57]
	SOD1^G93A^, TDP-43^Q331K^ and C9orf72-500 mice	GPX4 ↓ (total forebrain)	[58]
	SOD1^G93A^ mice	NRF2 ↓ (total forebrain)	[58]
Autophagy	SOD1^G93A^ mice	mTOR ↓, LC3 and p62 ↑ (total spinal cord), accumulation of autophagic vacuoles (motoneurons)	[71]
	SOD1^G93A^ mice	LC3 ↑ (spinal cord motoneurons)	[73]
	SOD1^G93A^ mice	increase in p62 aggregates and LC3B expression (motoneurons)	[74]
	sporadic ALS patient samples	LC3 ↑, autophagosomes ↑ (lumbar spinal cord motoneurons)	[77]
	SOD1^G93A^ mice	LC3 and p62 is unchanged (motor cortex)	[71]
	TDP-43^A315T^ mice	LC3 and p62 is unchanged (motor cortex)	[72]

The table summarizes the evidence indicating activation of cellular death pathways in ALS. These alterations are separated based on whether the quantification method was cell-type specific (directly indicating motoneuronal activation), or not (total spinal cord and motor cortex tissue lysate). *Bax* Bcl-2 associated X, *Bak* Bcl-2 homologous antagonist/killer *Bcl-2* B-cell lymphoma 2, *RIPK* receptor-interacting protein kinase, *Mlkl* mixed lineage kinase domain like pseudokinase, *OPTN* optineurin, *NLRP3* NOD-, LRR- and pyrin domain-containing protein 3, *GSDMD* gasdermin D, *GSDME* gasdermin E, *GSH* glutathione, *GPX4* glutathione peroxidase 4, *FTH1* ferritin heavy chain 1, *NRF2* NF-E2–related factor 2, *mTOR* mammalian target of rapamycin, *LC3* light chain 3.

## Necroptosis

Necroptosis is a form of regulated cell death (RCD), where the activation of certain cell-surface receptors (e.g., FAS, Tumor necrosis receptor 1 (TNFR1), Toll-like receptor 3/4 (TLR3, TLR4)) lead to the consequent activation of receptor-interacting protein kinase 3 (RIPK3) and mixed lineage kinase domain like pseudokinase (MLKL) [35, 36]. The phosphorylated MLKL (pMLKL) forms oligomers and binds to the plasma membrane, compromising its integrity (Fig. 1) [37].

In an in vitro model of ALS, human primary sporadic ALS astrocytes triggered the death of the co-cultured embryonic stem-cell-derived motoneurons in a caspase-independent manner. The RIPK1 antagonist necrostatin-1 prevented this motoneuronal loss [38], thus the authors concluded that ALS astrocytes induced motoneuronal necroptosis in their model. In a similar setup, silencing of *Ripk3* protected primary motoneurons when co-cultured with SOD1^G93A^ astrocytes [39]. Additionally, Ito et al. reported that the knockout of the ALS-linked *optineurin* (OPTN) gene in mice lead to dysmyelination and axonal degeneration through necroptotic mechanisms and the expression of RIPK1, RIPK3 and MLKL were increased in the spinal cord of *Optn*^-/-^ mice [40]. However, the authors also concluded that the necroptotic pathways were primarily activated in oligodendrocytes and the number of motoneurons in the spinal cord remained unaffected, not supporting motoneuronal necroptosis in this model of ALS. In the SOD1^G93A^ transgenic mouse model, the mRNA levels of *Ripk1*, *Ripk3* and *Mlkl* and the protein level of RIPK1 were reported to be elevated in the spinal cord, but the deletion of *Ripk3* failed to provide any functional or morphological benefits [39]. Other studies also confirmed the elevation of RIPK1 levels in the spinal cord but concluded that the genetic inactivation of *Ripk1* or *Mlkl* does not improve the disease progression or survival in SOD1^G93A^ mice [41, 42]. Fluctuating levels of the RIPK3-targeting miRNA-183-5p have been reported in SOD1^G93A^ spinal cord samples, as it was increased at the early symptomatic stage, while showing reduced expression in late symptomatic stage. Interestingly, these alterations were present in the spinal cord exclusively, as the cortical expression of miR-183-5p remained unaffected [43].

In human post-mortem spinal cord and spinal nerve samples, the level of phosphorylated RIPK (pRIPK)1 was only elevated in the endothelial cells of the spinal nerve, while no expression change was detected in the spinal cord of ALS patients [41], further discouraging the idea of motoneuronal necroptosis in ALS. Moreover, multiple papers have reported that the levels of RIPK1 remained unchanged in the motor cortex of SOD1^G93A^ mice [41, 42] and ALS patients as well [39]. Interestingly, increased levels of pMLKL was reported in the post-mortem hippocampus samples of ALS patients, whereas the authors could not detect any evidence of pRIPK1, pRIKP3 or pMLKL in the motor cortex of these patients [44].

Based on this data, while it was shown that ALS astrocytes induce motoneuronal necroptosis in vitro, there is no conclusive proof of motoneuronal or pyramidal cell necroptosis in in vivo models of ALS so far (Table 1). The genetic modulation of the necroptotic pathway also failed to provide any tangible benefit in the animal models of ALS, limiting its potential therapeutic applicability (Fig. 2). Nevertheless, a RIPK1 inhibitor (SAR443060) has been enrolled to a clinical trial, but was suspended due to toxicity issues [45].

## Pyroptosis

Pyroptosis is an inflammatory, caspase-dependent regulated cell death mechanism (Fig. 1). The canonical pyroptosis pathway is based on (i) the recognition of danger signals by inflammasome sensors (e.g., NOD-, LRR- and pyrin domain-containing protein 3 (NLRP3)), (ii) inflammasome assembly and (iii) the subsequent activation of caspase-1. In the non-canonical pathway, the upstream sensors are absent and caspase-4/5 is directly activated by intracellular lipopolysaccharide, making it primarily relevant in host defense. Both pathways converge on the main executioner protein, gasdermin D (GSDMD), which forms pores on the cell membrane upon activation [46]. Additionally, a caspase-3-dependent novel pathway was recently discovered, where the cleaved caspase-3 activates another member of the gasdermin family, gasdermin E (GSDME) [47].

NLRP3 inflammasome activation was detected in different cell types in various studies in the spinal cord of SOD1^G93A^ transgenic mice. Zhang et al. reported that NLRP3 was already detectable in the motoneurons at pre-symptomatic age, but later, in the symptomatic phase, also appeared in spinal cord astrocytes and microglia [48]. Somewhat controversially, Johann et al. detected NLRP3 inflammasome assembly in astrocytes in pre-symptomatic SOD1^G93A^ mice, but not in neuronal cells [49], while another study reported microglial NLRP3 activation in TAR-DNA binding protein 43 (TDP-43)^Q331K^ transgenic mice [50]. Interestingly, after acute traumatic nerve injury motoneurons were also the initial cell type of NLRP3 inflammasome assembly and activation in the spinal cord, while astroglial NLRP3 increase only occurred in a delayed manner [51]. Based on this, it is feasible that the cell-type specificity of the inflammasome assembly is very time-sensitive, which may even vary across different animal models. Nevertheless, it is clear that inflammasome assembly can happen in spinal cord motoneurons, enabling the activation of the canonical pyroptosis pathway. Indeed, increased GSDMD expression was reported in the spinal cord motoneurons of SOD1^G93A^ mice, while glial GSDMD expression was only detected at the late symptomatic disease phase [48]. In post-mortem spinal cord and motor cortex tissue of sporadic and C9orf72 ALS patients, increased caspase-1 and GSDMD expression was reported. Even though weak cleaved GSDMD signal was detected in neuronal cells in the motor cortex as well, the increased expression of pyroptosis proteins was primarily associated with microglial cells [52]. Interestingly, a recent study by Neel et al. reported increased levels of cleaved GSDME in spinal cord tissue of SOD1^G93A^ mice and the genetic ablation of *Gsdme* increased the lifespan and motoneuronal survival and improved the muscle strength in SOD1^G93A^ mice [53]. Furthermore, increased GSDME level was detected in primary mouse cortical neurons transfected with TDP-43, suggesting that pyroptosis might contribute to cortical neuronal cell death as well [53].

While the cell-type specificity of pyroptosis in the different models of ALS is somewhat controversial and requires further investigation, there is an increasing body of evidence suggesting that inflammasome activation and pyroptosis contribute to the pathomechanism of ALS (Table 1). The pyroptotic pathway provides various targets for potential interventional approaches in the future (e.g., NLRP3, inflammasome activation, caspase-1, GSDM family), however, as of now the effect of their modulation has remained mostly unexplored in ALS (Fig. 2).

## Ferroptosis

Ferroptosis is a recently discovered form of non-apoptotic, caspase-independent, regulated cell death (Fig. 1). It is triggered by the iron-dependent inactivation of antioxidant defense mechanisms, most likely through the inhibition of glutathione peroxidase 4 (GPX4) and depletion of intracellular glutathione (GSH) levels [54]. In the absence of antioxidant protection iron oxidizes membrane lipids, resulting in lipid peroxidation and consequent oxidative cell death [55]. In addition to these biochemical features, ferroptosis is accompanied by distinct morphological alterations as well, mostly affecting the mitochondrial structure (i.e., shrinkage of mitochondria, reduction, and disappearance of the mitochondrial cristae, increase in the density of mitochondrial membranes) [56].

Multiple studies indicate that cell death via ferroptosis is directly involved in the pathomechanism of ALS. Yang et al. reported that the level of GSH declined in spinal cord and motor cortex samples of SOD1^G93A^ mice, in parallel with disease progression. In the same study, transmission electron microscopy revealed morphological alterations characteristic of ferroptosis in spinal cord motoneurons [57]. The other key component of the ferroptotic pathway, GPX4 showed reduced expression in spinal cord and brain samples of SOD1^G93A^, TDP-43^Q331K^ and C9orf72-500 mouse models of ALS [58]. Moreover, ferritin heavy chain 1 (FTH1) and NF-E2–related factor 2 (NRF2) levels were downregulated in the spinal cord and forebrain of SOD1^G93A^ mice, further supporting iron metabolism dysregulation [58]. Post-mortem analysis of spinal cord samples of sporadic and familial ALS patients confirmed the presence of similar alterations (i.e., GPX4 depletion) [58], while another study reported iron accumulation in spinal cord motoneurons of sporadic ALS patients [59].

Based on these findings, ferroptosis seems to be an important component of ALS pathomechanism, and various studies examined whether the modulation of the pathway impacts disease progression. Chen et al. found that overexpression of *GPX4* preserves the integrity of spinal cord motoneurons, delays disease onset and prolongs the lifespan of SOD1^G93A^ mice [60], which was later corroborated by others as well [61]. Ferrostatin-1, a selective inhibitor of ferroptosis, also exerted beneficial effects on both motoneuron loss and motor performance of SOD1^G93A^ mice [61]. Finally, administration of an iron-chelator ferroptosis inhibitor drug, VAR10303, in combination with calorie-enriched diet reduced motoneuron loss, improved motor endplate innervation and prolonged the lifespan of SOD1^G93A^ mice [62].

These findings support the importance of ferroptosis in motoneuronal and pyramidal cell death, based on both murine and post-mortem patient samples (Table 1). The genetic and pharmacological modulation of the ferroptotic pathway also yielded promising preclinical results, however, these reports were all limited to the SOD1^G93A^ mouse model of ALS, thus additional studies are required to further evaluate their beneficial effects (Fig. 2).

## Autophagy

Autophagy is a highly conserved degradation pathway that is responsible for the lysosome-mediated breakdown and regulated turnover of intracellular components. The process occurs virtually in all living cells at low basal levels, and can be further activated upon various stimuli, including nutrient deprivation, protein aggregates, misfolded proteins, as well as damaged organelles [63]. Although under physiological conditions autophagy plays a fundamental role in cell survival, dysregulation of the autophagic machinery can culminate in caspase-independent cell death (Fig. 1) [64].

Among the three main types of autophagy (i.e., macroautophagy, microautophagy and chaperon-mediated autophagy), macroautophagy is considered to be the most universal mechanism, generally characterized by the formation of double-membrane structures called autophagosomes that deliver cytoplasmic constituents to lysosomes for degradation [65]. The induction of macroautophagy is tightly controlled by various regulatory molecules, among which mammalian target of rapamycin (mTOR) plays a fundamental role as a negative regulator. Other regulators, such as histone deacetylases (HDAC), including HDAC6 can modulate autophagic activity on multiple levels (e.g., post-translational modifications of autophagy-related factors, transportation and degradation of autophagosomes), representing a more complex regulatory effect [66, 67]. Once activated, the progression of autophagy relies on the presence of “core” autophagy complexes, containing various autophagy related (e.g., Atg) proteins [68]. Their conjugation facilitates the buildup and elongation of phagophores, forming autophagosomes. One of the key events of autophagy is the processing of microtubule‑associated protein 1 A/1B-light chain 3 (LC3) to its active form, LC3-II, which has a prominent role in the maturation of the phagophores, as well as in the selection of cargo to be degraded [69]. Another key event, necessary for the execution of autophagy is the fusion of autophagosomes with lysosomes yielding autolysosomes containing degradative enzymes that catabolize the engulfed constituents [63]. The increase in autophagy-related protein levels (e.g., LC3-II, Atg proteins) is indicative of upregulated autophagosome formation, however, in certain conditions it may represent suppressed autophagic degradation [70]. Therefore, to fully understand the role of autophagy in the pathomechanism of various diseases, autophagosome-related findings must be carefully assessed, and potentially corroborated via the detection of regulatory proteins and presence of autolysosomes, as well as by the investigation of the effects of autophagy modulators.

Multiple studies investigated the role of autophagy in the pathomechanism of ALS, especially its contribution to the ALS-related proteinopathy. Zhang et al. reported mTOR-dependent increase in LC3-II and p62 levels in the spinal cord of SOD1^G93A^ mice, which seemed to be a motoneuron-specific alteration. Additionally, they found no autophagy-related alterations in the motor cortex, suggesting that increased autophagic flux is limited to the spinal cord motoneurons [71]. This was further corroborated by Gou et al., as they reported that the levels of LC3 and p62 remained unchanged in the motor cortex of TDP-43^A315T^ mice [72]. Tian et al. monitored the activity of autophagy at different stages of ALS (i.e., presymptomatic, early and late symptomatic stages) in SOD1^G93A^ mice in vivo, revealing that the level of LC3 considerably increased in spinal cord motoneurons during early and late symptomatic phases. Interestingly, these alterations were not motoneuron-specific, as they were detectable in the surrounding microglia and astrocytes as well [73]. Rudnick et al. further examined whether autophagy has a causative role in ALS by crossing Atg7 conditional knockout mice to the SOD1^G93A^ mouse model of ALS. Their findings indicated that inhibition of autophagy accelerates disease onset, most likely due to faster endplate denervation. Although the deletion of Atg7 did not affect motoneuron cell death, it interestingly prolonged the survival of double-mutant mice, suggesting that autophagy might have dual role in ALS (i.e., it might be protective against motoneuron degeneration in early phases, while being an important pathogenic feature at later stages) [74].

Among various autophagic modulators, HDAC6 was shown to have decreased levels in the spinal cord of presymptomatic SOD1^G93A^ mice [75]. The authors also concluded that the altered HDAC6 expression contributes to motoneuronal degeneration, as the overexpression of HDAC6 delayed motoneuron loss via enabling autophagolysosome formation and prolonged the lifespan of SOD1^G93A^ mice [75]. Somewhat controversially, Fazal et al. has demonstrated that treatment of *TARDPB*-mutant induced pluripotent stem cell (iPSC)-derived human spinal motoneurons with a HDAC6 inhibitor reverted TDP-43 pathology and maintained motoneuronal integrity [76]. These findings showcase the highly complex and time-dependent manner of autophagic modulation in various models of ALS.

A post-mortem analysis revealed that signs of autophagy induction are present in lumbar spinal cord motoneurons of sporadic ALS patients [77]. Furthermore, the number of autophagosomes and autolysosomes was higher in patients who had shorter clinical course and relatively well-preserved ventral horn motoneurons, suggesting a protective role of autophagy in early stages of ALS [77]. Moreover, post-mortem analysis of human spinal cord, brainstem and motor cortex samples revealed that the loss of function mutation of *TARDP* results in abnormal alternative splicing of *ATG4B* transcripts, ultimately culminating in impaired autophagy [78]. The altered expression of the regulatory HDAC family was also implicated in ALS patients, as the post-mortem analysis of brain and spinal cord samples revealed decreased *HDAC2* and increased *HDAC11* mRNA levels [79]. Nevertheless, their exact role in regulating autophagy and other cell death mechanisms currently remain unknown.

Since autophagy seems to have an important role in ALS, multiple studies analyzed the effects of autophagy modulation on disease progression. The effects of rapamycin, a well-known autophagy inducer, were evaluated in various experimental setups. The analysis of the effects of rapamycin treatment revealed that the drug may have beneficial effect in presymptomatic stages, as it delayed the disease onset significantly in transgenic SOD1^G93A^ mice [80]. However, at later stages rapamycin was found to accelerate the progression of ALS, eventually resulting in a shorter lifespan [80, 81]. Somewhat controversially, Zhang et al. reported that rapamycin treatment resulted in earlier disease onset in SOD1^G93A^ mice [71], contradicting its potential protective role in presymptomatic disease stage. Since it has also been demonstrated that certain proteins, including mutant SOD1, may facilitate the development of rapamycin resistance [82], it has been proposed that the effects of the drug should be tested in additional models of ALS. Although rapamycin treatment has not been investigated in other in vivo models of ALS so far, an in vitro model using motoneurons differentiated from embryonic stem cells isolated from TDP‑43^A315T^ mice reported neuroprotective effects [83]. The beneficial effects of autophagy induction were confirmed by Kim et al., who reported that progesterone treatment reduced spinal cord motoneuron cell death, delayed the progression of symptoms, and prolonged the survival of SOD1^G93A^ mice, which effects were diminished by the inhibition of autophagy by the administration of 3-methyladenine [84].

The potential role of autophagy suppression has been tested as well in ALS. Hsueh et al. reported that n-butylidenephthalide (nBP) treatment of SOD1^G93A^ mice downregulated the autophagic activity in the spinal cord, as revealed by increased level of autophagy repressor mTOR, and decreased expression of Atg5 and LC3-II. Moreover, nBP treatment improved motor function, reduced the loss of spinal cord motoneurons and extended the lifespan [85]. It has also been demonstrated that administration of Trehalose, a natural disaccharide that induces mTOR-independent autophagy, delayed disease onset, improved the survival of ventral horn motoneurons and increased the lifespan of SOD1^G86R^ mice [86].

Despite the controversies in preclinical studies, autophagy induction and immunosuppression by rapamycin was investigated in ALS patients who do not carry SOD1 mutations. The randomized, double-blind, placebo-controlled trial reported safe applicability of low-dose rapamycin in ALS patients, however, the drug’s effect on clinical outcome could not be quantified [87]. Another phase II/III clinical trial is ongoing which evaluates the beneficial effects of SLS-005 Trehalose in ALS patients (NCT04297683).

Autophagic dysregulation has been established as a key component of motoneuronal degeneration in ALS (Table 1). However, it might have a dual role, as in early stages of the disease autophagic flux can exert a protective role through degradation of protein aggregates, while facilitating motoneuronal death in later stages. This might explain, at least partially, the contradictory findings that emerged across the various studies on autophagy modulation (Fig. 2). Moreover, autophagic cell death is interconnected with multiple alternative cell death pathways, hence treatments modulating autophagic activity may influence other cell death pathways as well (Fig. 3). Further investigation of the autophagy-related cell death mechanisms is needed to clarify the exact role and time-dependent quality of autophagic machinery in ALS.

## Conclusion and future perspectives

Motoneuronal loss is considered to be the center piece of ALS pathophysiology. Human autopsy studies indicate that 20% of spinal cord motoneurons are lost before the onset of the symptoms [88], thus cell death mechanisms are already activated in the early stage of the disease. While it was originally hypothesized that apoptosis is the major pathway leading to motoneuronal death in ALS [10], the contributory role of necroptosis, pyroptosis, ferroptosis and autophagy have shifted into the center of focus recently. These investigations often yielded seemingly contradictory findings, however, the following factors should be considered, when evaluating these results: (a) the majority of these studies were based on the SOD1^G93A^ mouse model, however, different (e.g., TDP-43- and C9orf72-based) mouse and iPSC-based models might provide different findings and shed light on additional/alternative pathways; (b) the timing of the investigation (i.e., presymptomatic, early symptomatic or late symptomatic) will drastically impact the findings, as seen in the case of pyroptosis and autophagy; (c) these cell death mechanisms might be activated in a single (i.e., neuronal cells) or multiple cell types in parallel. While in this review we are focusing on the above-mentioned cell death pathways, additional mechanisms should be considered to contribute to motoneuronal demise as well. For instance, the activation of parthanatos was also implicated in murine ALS models and ALS patient spinal cord samples [89, 90], however very limited information is available on these further cell death pathways. Furthermore, there is a complex interplay of various pathophysiological processes in ALS, including, but not limited to protein aggregation, inflammation, oxidative stress, mitochondrial dysfunction, and calcium disbalance [91]. These processes can induce different cell death mechanisms, leading to the activation of multiple cell death pathways in parallel, adding a further layer of complexity to understanding motoneuronal degeneration (Fig. 3). Nevertheless, there is clear evidence that apoptotic, ferroptotic, pyroptotic and autophagy-related cell death mechanisms are activated in spinal cord motoneurons during the course of the disease, while the current evidence does not support the activation of necroptosis in these cells. In the motor cortex, reports indicate the activation of apoptosis, ferroptosis and possibly pyroptosis in pyramidal cells, while necroptosis and autophagy-related cell death seemingly do not contribute to neuronal loss.

The genetic and pharmacological modulation of cell death pathways have resulted in promising preclinical findings; however, it is yet to be seen if these translate into the clinical practice. A limited number of clinical trials have been conducted or are currently underway with pharmacological agents targeting cell death pathways (e.g., RIPK1 inhibitor, Rapamycin, Trehalose), however, these have remained mostly unconclusive [92]. The parallel activation of different cell death mechanisms also raises the need for evaluating the efficacy of combined therapies, modulating multiple pathways at the same time. Furthermore, understanding the interplay between these different pathways might further improve our understanding of motoneuronal pathology in ALS. While motoneuronal and pyramidal cell death were considered to be an inevitable endpoint of ALS pathology, a growing body of evidence confirmed that these downstream pathways might still be utilised for therapeutic applications.
